# 96 Poly-dl-lactide Copolymer-dressing Use on Burn Wounds and Skin Graft Donor Sites - An Institutional Review

**DOI:** 10.1093/jbcr/irac012.098

**Published:** 2022-03-23

**Authors:** Tomer Lagziel, Garyn Metoyer, Qingwen Kawaji, Arya A Akhavan, Carrie A Cox, Julie Caffrey, Charles S Hultman, Eliana F Duraes

**Affiliations:** Johns Hopkins University School of Medicine / Sackler School of Medicine, New-York Program, Tel-Aviv University, Rockville, Maryland; Sinai Hospital of Baltimore, Baltimore, Maryland; Johns Hopkins, Baltimore, Maryland; Johns Hopkins Hospital, North York, Ontario; Johns Hopkins Bayview Medical Center, Baltimore, Maryland; Johns Hopkins, Baltimore, Maryland; Johns Hopkins University School of Medicine, Baltimore, Maryland; Johns Hopkins University School of Medicine, Baltimore, Maryland

## Abstract

**Introduction:**

In burn surgical care, wound coverage and the corresponding dressing are paired to maximize the ability to promote re-epithelization, minimize pain and patient discomfort, dressing change frequency and overall cost. This dressing, a copolymer material based on DL lactic acid, has been described as a reliable alternative dressing for partial thickness burns as well as skin graft donor sites with comparable wound-healing quality and duration. Our aim is to assess outcomes results of this copolymer dressing at our institution, as applied to partial thickness burn wounds and graft donor sites.

**Methods:**

We performed a retrospective analysis of 55 adult patients admitted between January 1, 2020 to August 25, 2021 for the treatment of partial thickness burns that were managed with a poly-DL-lactide copolymer skin substitute at the burn wound and/or autograft donor site. Three study groups were established based on application site: wound only (group 1), donor site only (group 2), and both (group 3). We assessed operative times, infections rates, complications, length of stay, readmission rates, and mortality.

**Results:**

Preliminary data of 40 patients shows clinically similar results for analgesic requirements, operative length, and hospital LOS between group 1 and group 3. Group 2 showed higher analgesic requirements, lower operative times, a lower LOS, and lower readmission rates. Group 3 shows higher pain levels and longer operative times, when compared with groups 1 and 2, but lower readmission rates than group 1.

**Conclusions:**

The poly-DL-lactide copolymer skin substitute offers reliable wound coverage for a partial thickness burns while also reducing frequency of dressing changes and associated pain correlating to reduced length of hospital stay and wound healing interval.

